# Commentary: Could We Address the Interplay Between CD133, Wnt/β-Catenin, and TERT Signaling Pathways as a Potential Target for Glioblastoma Therapy?

**DOI:** 10.3389/fonc.2021.712358

**Published:** 2021-08-12

**Authors:** Christine A. Fargeas, Aurelio Lorico, Denis Corbeil

**Affiliations:** ^1^Tissue Engineering Laboratories, Biotechnology Center (BIOTEC) and Center for Molecular and Cellular Bioengineering, Technische Universität Dresden, Dresden, Germany; ^2^College of Medicine, Touro University Nevada, Henderson, NV, United States

**Keywords:** CD133, cilium, prominin-1, membrane vesicle, Wnt/β-catenin, glioblastoma

We do not intend to analyze here the meaning of the title of the article “Could We Address the Interplay Between CD133, Wnt/β-Catenin, and TERT Signaling Pathways as a Potential Target for Glioblastoma Therapy?” by Behrooz and Syahir published recently in Frontiers in Oncology [issue April 1, 2021]. Yet, it is important to mention that essential data regarding the biology of prominin-1 (prom1, a.k.a. CD133) are missing and the work of others is mistakenly described. We here provide these quotations to guide the reader to the literature pertinent to the question raised.

Targeting prominin-1-expressing cells in relation to signaling cascades for cancer therapy has indeed been the concern of many over the past decade. To fully apprehend the clinical potential of targeting strategies, it is important to provide the complete and accurate account of the biology of prominin-1. Initially identified as a marker for plasma membrane protrusions in mouse embryonic neuroepithelial cells, the primary neural stem cells (1), prominin-1 has been used to identify and purify murine stem cells from the adult subventricular zone along the lateral ventricle walls, hippocampal dentate gyrus, and the postnatal cerebellum, which has important implications for the comprehension of cerebellar development and the origins of medulloblastoma (2–5). Its human homolog (6) was identified on a subpopulation of CD34^+^ hematopoietic stem cells (7) and characterized from retinoblastoma cell lines (8). Human neural precursors were shown to harbor prominin-1 (9), which was later used to identify and purify brain tumor stem cells (10). Since then, prominin-1 has been considered as a prominent marker associated with tumor development in the field [see the review (11) and references therein].

An interconnection between prominin-1 and canonical Wnt pathway was initially suspected in melanoma cells several years ago (12). The downregulation of prominin-1 prevented the nuclear localization of β-catenin and reduced Wnt signaling through TCF/LEF transcription factor (13). Yet, the Wnt/β-catenin pathway could still be activated by its physiological ligands as nuclear localization of β-catenin was restored upon addition of Wnt3a to prominin-1-knockdown cells (13). TCF-LEF-binding sites are present in the *PROM1* gene promoter (14). Interaction of prominin-1 with histone deacetylase 6 was shown to stabilize β-catenin in a colon carcinoma cell line (15), and prominin-1 was suggested to mediate activation of the phosphoinositide 3-kinase (PI3K)/protein kinase B (AKT) pathway in glioma stem cells by its binding to p85 regulatory subunit of PI3K (16). The interplay between these pathways and others in carcinogenesis was discussed in a Letter to the Editor entitled “*An intriguing relationship between lipid droplets, cholesterol-binding protein CD133 and Wnt/β-catenin signaling pathway in carcinogenesis”* (17).

In the context of brain cancer, it is essential to note that many cells in the adult central (and peripheral) neural systems, other than cells with stem cell properties (i.e., self-renewal and differentiation capacities) that reside in the subventricular zone or subgranular zone of the hippocampus, express prominin-1 (18–21). Although the expression profile appears to be different in humans (21), the unrestricted expression of prominin-1 by multiple cell types including multiciliary ependymal cells and other glial cells tend to indicate that its association with stem cells properties is not absolute [reviewed in (22)]. Indeed, prominin-1 is detected within white matter tracts of postnatal and adult brain (21, 23), where it is expressed by a subset of cells within the oligodendrocyte lineage and is a constituent of myelin sheaths (23, 24). Consistently, its expression is drastically reduced in the brain of myelin-deficient mice (23), and hypomyelination and cognitive impairment were observed in mice lacking prominin-1 (24). Prominin-1 is also detected in a subgroup of astrocytes (B1 type) that are in contact with the lateral ventricle, including those that act as quiescent or activated neural stem cells in adults (3, 25). There, prominin-1 concentrates at the tip of the primary cilia of the quiescent stem cells and redistributes across the apical surface of the activated ones that are devoid of primary cilium. It remains to be established whether prominin-1 itself is somehow involved in the stem cell activation process therein, as recently demonstrated in mouse dental stem cells (26). Interestingly, in proneural glioblastoma-like tumor cells, prominin-1 is expressed by the endothelium that supports microvascular proliferation and accelerates tumor growth (20). On the other hand, several studies concluded that prominin-1 expressing cells were dispensable for gliomagenesis (18, 27, 28). Holmberg-Olausson and colleagues provided evidence that human prominin-1 may constitute a tumor cell-intrinsic marker independent of stem cell properties, as they observed a variability of prominin-1 expression levels in human glioblastoma, where higher prominin-1 expression correlated with shorter patient survival (21). This differential expression of prominin-1 may be reflected in the amount of prominin-1^+^ membrane vesicles found in the cerebrospinal fluid of glioblastoma patients (29) (see below).

The presence of distinct prominin-1 splicing variants in neural stem cells, oligodendrocytes, and astrocytes, which suggests related but distinct functions (5, 23, 30), adds complexity to the analysis of the origin of neural cancer cells notably by immunohistochemistry, as prominin-1 splice variants may differentially mark cell subpopulations in glioma. Besides, the detection of human prominin-1 can be altered by the use of certain anti-prominin-1 antibodies whose corresponding epitopes may be masked under native conditions (31). These issues have led to the generation of contradictory data and discussions and should be kept in mind when addressing prominin-1 in normal and pathological conditions such as brain cancer.

In addition to cellular components, it is important to describe that prominin-1 is released in association with membrane vesicles into the cerebrospinal fluid (32), and their amount is elevated in glioblastoma patients (29), which could impact the surrounding cells in the cancer microenvironment and beyond. Prominin-1^+^ vesicles, budding from microvilli and/or primary cilia, could be the source of signals between cells as they facilitate the exchange of active molecules (33). In cancer, some studies suggest that prominin-1 is also associated with exosomes (13, 32, 34) as demonstrated in blood stem cells (35). It remains to be demonstrated whether these prominin-1^+^ membrane particles help in cell differentiation, as Behrooz and Syahir wrote. In other words, it is not completely clear whether the differentiation process is induced by the loss of prominin-1^+^ vesicles or whether their release is a consequence of differentiation. Data suggest that the former may occur in colon cancer (32, 34). In addition, it is worth mentioning that prominin-1 has been shown to segregate symmetrically/asymmetrically during cell division in glioma stem cells in the presence or absence of growth factors, which contributes to the maintenance of the cancer stem cell pool in the tumor and its cellular heterogeneity (36). As a result, the context of signaling would differ in the different cell progeny. Importantly, targeting prominin-1 might, beyond impacting prominin-1 expressing cells, also alter prominin-1^+^ vesicle-mediated intercellular communication within the cancer stem cell microenvironment.

The authors present prominin-1 as a surface receptor, citing their previous review in *J. Drug Target* [issue March 2019], and prominin-1, Wnt/β-catenin, and TERT as three signaling cascades. Recently, a prominin-1–AKt–Wnt signaling axis in glioblastoma tumor-initiating cells was indeed described, leading to the hypothesis that prominin-1 may act as a putative surface receptor (37). Yet, to the best of our knowledge, no extracellular ligand, the binding of which would generate signal transduction, was described for prominin-1. This molecule is involved in various protein–protein and lipid–protein interactions notably those regulating the architecture and dynamics of filopodia, microvilli, and cilia (26, 38, 39). Behrooz and Syahir wrote that “*the rigorous function of CD133 maintains unidentified, but it has been proposed that it would perform as cell membrane topology organizer*”. However, they did not cite any reference nor developed this aspect. We therefore recommend the following review for further reading on this topic (40). Indeed, as the primary cilium is an important signaling hub during development and in mature brain, and numerous ciliary-associated genes are dysregulated in cancer, the implication of prominin-1 in the dynamics of primary cilium might potentially account, at least partially, for its interplay with several signaling pathways found therein, including Wnt/β-catenin (41). In this regard, it is of note that the absence of prominin-1 as an organizer of plasma membrane protrusions impairs the general configuration of ependymal cilia, and potentially their functions such as mediating the circulation of cerebrospinal fluids necessary for brain homeostasis and delivering signaling molecules (42). Thus, prominin-1 could be considered a marker for cells with high plasma membrane dynamics and turnover, including cells with high proliferative potential and self-renewal capacity. The implication of prominin-1 in signaling cascades related to carcinogenesis needs to be further dissected. Nevertheless, prominin-1 remains promising, as a cell surface marker, for therapeutic development.

## Author Contributions

CF, AL, and DC designed and wrote the manuscript. All authors contributed to the article and approved the submitted version.

## Conflict of Interest

The authors declare that the research was conducted in the absence of any commercial or financial relationships that could be construed as a potential conflict of interest.

## Publisher’s Note

All claims expressed in this article are solely those of the authors and do not necessarily represent those of their affiliated organizations, or those of the publisher, the editors and the reviewers. Any product that may be evaluated in this article, or claim that may be made by its manufacturer, is not guaranteed or endorsed by the publisher.
